# Involvement of Fatty Acids and Their Metabolites in the Development of Inflammation in Atherosclerosis

**DOI:** 10.3390/ijms23031308

**Published:** 2022-01-24

**Authors:** Stanislav Kotlyarov, Anna Kotlyarova

**Affiliations:** 1 Department of Nursing, Ryazan State Medical University, 390026 Ryazan, Russia; 2 Department of Pharmacology and Pharmacy, Ryazan State Medical University, 390026 Ryazan, Russia; kaa.rz@yandex.ru

**Keywords:** atherosclerosis, inflammation, innate immunity, hemodynamics, lipids, fatty acids, eicosanoids

## Abstract

Despite all the advances of modern medicine, atherosclerosis continues to be one of the most important medical and social problems. Atherosclerosis is the cause of several cardiovascular diseases, which are associated with high rates of disability and mortality. The development of atherosclerosis is associated with the accumulation of lipids in the arterial intima and the disruption of mechanisms that maintain the balance between the development and resolution of inflammation. Fatty acids are involved in many mechanisms of inflammation development and maintenance. Endothelial cells demonstrate multiple cross-linkages between lipid metabolism and innate immunity. In addition, these processes are linked to hemodynamics and the function of other cells in the vascular wall, highlighting the central role of the endothelium in vascular biology.

## 1. Introduction

Atherosclerosis is one of the key medical and social problems of modern society [1]. Coronary heart disease, cerebral stroke and peripheral arterial disease, which develop with atherosclerosis, are associated with high rates of hospitalizations and temporary and permanent disability, and are among the leading causes of mortality in many countries [2,3,4]. A better understanding of the mechanisms of atherosclerosis and the factors contributing to its progression is therefore an important challenge.

Although many aspects of atherogenesis have been intensively studied, there are still many unanswered questions. The complexity of the problem of atherosclerosis stems from the fact that its development in patients takes place over many years and that there are no available clinical tools that can determine its onset. The development of atherosclerosis is thought to be closely related to lifestyle, including diet, especially dietary fats and physical activity levels [5]. Modification of these risk factors plays an important role in providing effective therapy [6,7].

Throughout the long history of the study of atherosclerosis, the role of lipids has received increased attention. The results of numerous studies highlight the diverse functions of lipids in atherogenesis.

According to modern concepts, atherosclerosis is considered as a disease characterized by excessive accumulation of lipids in the arterial intima, in which the balance of mechanisms associated with the development and resolution of inflammation is disturbed. Atherosclerosis is thought to begin with the accumulation of lipoproteins containing apolipoprotein B in the arterial intima, accompanied by endothelial cell activation, recruitment of leukocytes, particularly monocytes, and leads to the accumulation of cells, extracellular matrix and lipids in the arterial intima [8].

Atherosclerotic plaque formation has a complex pathogenetic mechanism. Its development represents a conditional staging process, which emphasizes the dynamic nature of the disease. Early development of fatty streaks is associated with increased transcytosis of low-density lipoprotein (LDL) particles, an inflammatory response involving the innate immune system, the formation of foam cells, and the formation and fusion of extracellular lipid pools. Early fibroatheroma is characterized by disruption of the normal architecture of the intima. Accumulation of extracellular lipids and cellular necrosis lead to attachment of fibrous tissue, which forms a fibrous cap over the lipid-rich necrotic cores. The activity of proteolytic enzymes causes the fibrous cap to become thin and loose in several places, corresponding to the development of a thin-cap fibroatheroma. The thin cap is susceptible to rupture and is associated with thrombosis. The progression of atherosclerotic lesions is also characterized by calcium deposition [9].

The development of atherosclerosis has been shown to be caused by a complex of factors, both general and local in nature. Dyslipidemia, oxidative stress and systemic inflammation play a known role in the pathogenesis of atherosclerosis. However, they do not determine the specific localization of atherosclerotic lesions in certain parts of the arteries. In this regard, local factors such as local hemodynamic disturbances, endothelial cell activation and dysfunction are also important events in atherogenesis [10,11].

The potential role of mitochondrial deoxyribonucleic acid (DNA) mutations is also of interest. The presence of atherosclerosis-associated mitochondrial DNA mutations correlated with proinflammatory monocyte activation in patients with asymptomatic atherosclerosis [12]. Given the significant role of LDL in the pathogenesis of atherosclerosis, the role of LDL chemical modification seems important. Accumulated experience has shown that desialylation of lipoproteins is associated with their increased atherogenicity. LDL desialylation causes uncontrolled lipid accumulation by arterial cells [13].

A growing body of evidence reinforces the understanding that lipids are involved in many mechanisms of atherogenesis not only as a substrate, but also as important participants in many links in the complex chain of processes leading to atherosclerosis.

The purpose of this review is to discuss the cross-linkages in which long-chain fatty acids and their metabolites are involved in the development of inflammation in atherosclerosis. An analysis has been made of how endothelial function is related to fatty acids in atherosclerosis.

## 2. The Role of Fatty Acids in the Development of Inflammation in Atherosclerosis

Lipids are a heterogeneous group of chemicals in which fatty acids play an important role. Fatty acids are carboxylic acids with long aliphatic chains containing a methyl group at one end, while a carboxylic group at the other end. Depending on the length of the carbon chains fatty acids are divided into short-chain, medium-chain and long-chain fatty acids, which are designated as SCFAs, MCFAs and LCFAs, respectively. In addition, it is important to classify fatty acids according to the presence of double bonds. This classification includes saturated fatty acids (SFAs) without double bonds, monounsaturated fatty acids (MUFAs) with only one double bond and polyunsaturated fatty acids (PUFAs) with at least two double bonds [14].

The involvement of fatty acids and their lipid metabolites in cross-linking with inflammation in atherosclerosis is of interest. Fatty acids are involved in atherogenesis in different ways, demonstrating both pro- and anti-atherogenic functions. They may contribute to inflammation and endothelial dysfunction by participating in many signaling pathways [14,15].

Saturated fatty acids are thought to be associated with proinflammatory effects, which are provided by modifying the structure of the plasma membrane of cells, direct stimulation of proinflammatory signaling pathways. At the same time, PUFAs demonstrate a more complex role in inflammation. Accumulating evidence suggests a different contribution of ω-3 and ω-6 PUFAs to inflammation [16].

PUFAs can influence plasma membrane fluidity and lipid raft stability and act as a substrate for the biosynthesis of lipid mediators involved in inflammation regulation. For example, arachidonic acid, which belongs to ω-6 PUFAs, is a substrate for the biosynthesis of prostaglandins (PG) and leukotrienes (LT), which are involved in the initiation of inflammation [17,18]. However, arachidonic acid can also be used to biosynthesize lipoxin A4 (LXA4), which is considered an important participant in the resolution of inflammation [19,20,21,22]. In turn, the anti-inflammatory properties of ω-3 PUFAs are the subject of clinical trials aimed at finding new drugs that positively affect lipid metabolism and atherogenesis.

The involvement of fatty acids in atherogenesis is related to their diverse role in the function of many cells in the vascular wall, such as endothelial cells.

## 3. The Role of Endothelial Cells in Inflammation

Endothelial cells perform many functions to maintain normal hemodynamics [23,24]. Although their key role is considered to be the creation of a barrier between blood and surrounding tissues through dense intercellular contacts, as well as regulation of hemodynamics by the production of biologically active mediators, the accumulated evidence to date suggests that the role of endothelial cells in vascular biology is not limited to this [25]. Participation in the regulation of functional activity of other cells, both in the bloodstream and in the vascular wall, such as leukocytes or vascular smooth muscle cells, as well as participation in the immune response, are other important functions of the endothelium [26]. Studies of these functions contribute to a more detailed interpretation of the processes that occur in the vascular wall and which can be disrupted in atherosclerosis.

Indeed, the involvement of endothelial cells in immune function has been confirmed by a number of studies. Extensive data have previously been reported summarizing the information that endothelial cells perform a number of innate immune functions. These functions include cytokine secretion, pro-inflammatory, anti-inflammatory, phagocytic functions, antigen presentation, sensing of pathogen-associated molecular patterns (PAMPs) and damage-associated molecular patterns (DAMPs), immune enhancing and immunosuppressive function, cell migration, heterogeneity and endothelial cell plasticity [27,28].

Fatty acids are involved in some endothelial cell functions [29]. The phospholipid composition of plasma membranes depends on the external fatty acid input. The inclusion of saturated or unsaturated fatty acids in phospholipids is associated with effects on membrane biophysical properties and membrane protein function. Exogenous and endogenous polyunsaturated fatty acids are also involved in the formation of lipid droplets in endothelial cells, which may influence endothelial stiffness [30].

The study of cross-links between immune and metabolic processes has become an important new area of research in recent years. These findings are well demonstrated in macrophages, whose immunometabolic polarization is closely related to the function of these cells in inflammation. The pro-inflammatory M1 phenotype of macrophages mainly depends on glycolysis and fatty acid synthesis, whereas anti-inflammatory macrophages (M2 phenotype) prefer fatty acid oxidation, using exogenously produced fatty acids for this purpose [31,32,33,34]. In turn, endothelial cells both at quiescence and during inflammation or stimulation by vascular endothelial growth factor (VEGF) rely on glycolysis as a key energy source [35,36]. The β-oxidation of fatty acids is used for the de novo synthesis of deoxyribonucleotides, which are essential for endothelial cell proliferation [35,37]. β-oxidation of fatty acids is also essential for the maintenance of the tricarboxylic acid cycle in endothelial cells [38]. Fatty acid oxidation has been shown to be involved in redox homeostasis of endothelial cells. Carnitine palmitoyltransferase 1A (CPT1A) inhibition increased reactive oxygen species levels and resulted in decreased expression of antifibrinolytic genes, increased vascular wall permeability, and increased leukocyte adhesion and infiltration [38].

In addition, CPT1A inhibition experiments showed that fatty acid oxidation is a central regulator of endothelial cell permeability in vitro and blood vessel stability in vivo [39].

Fatty acid synthesis is known to play an important role in endothelial cell function. Endothelial cells deficient in fatty acid synthesis demonstrated insufficient migratory capacity and impaired angiogenesis [40]. It is suggested that palmitate derived from fatty acid synthesis can be used to protein palmitoylation related to the provision of immune functions and hemodynamics.

In addition to using fatty acids, endothelial cells regulate fatty acid transport to metabolically active tissues [41]. CD36 in endothelial cells plays an important role in the translocation of long-chain fatty acids from the circulation to the tissues.

Free fatty acids induce endothelial dysfunction by down-regulating the AMPK/ PI3K/Akt/eNOS signaling pathway [14]. Free fatty acids may contribute to inflammation leading to increased endothelial permeability [42]. Increased plasma levels of free fatty acids increased plasma markers of endothelial activation inter-cellular adhesion molecule 1 (ICAM-1) and vascular cell adhesion molecule 1 (VCAM-1) [43]. In an experiment with human adipose microvascular endothelial cells (HAMECs), palmitate was shown to increase the expression of interleukins (IL)-6, IL-8, Toll-like receptor 2 (TLR2), and ICAM-1. Palmitate-induced surface expression of ICAM-1 promoted monocyte binding and transmigration [44].

Thus, fatty acids demonstrate the involvement in the regulation of some parts of the innate immune system, which has significance in atherogenesis.

## 4. The Role of the Innate Immune System in Atherogenesis

A growing body of evidence supports the importance of the innate immune system in atherogenesis. The innate immune system is universal and highly evolutionarily conservative [45]. Recognition of PAMPs and DAMPs by immunocompetent cells is a crucial function of innate immunity. The innate immune system relies on a large family of pattern recognition receptors (PRRs), such as Toll-like receptors (TLRs), as the first line of defense of the organism [46,47]. TLR signaling pathways can be activated by various exogenous as well as some endogenous molecules, which may play an important role in the development and progression of atherosclerosis [48].

Increased expression of some TLRs in arterial endothelial cells in the area of atherosclerotic lesions has been shown. This increased expression correlated with cell activation [49]. Although the potential role of TLR4 in the early stages of atherogenesis remains a subject for study, it is known that the low expression of TLR4 in endothelial cells of normal arteries is markedly increased in atherosclerosis [50]. Activated endothelial cells are also characterized by increased production of IL-6, IL-8 and MCP-1 (monocyte chemoattractant protein-1) via TLR4 [51]. The results show that TLR4 may be involved in different stages of atherogenesis, from participation in activation of cell adhesion to enhancement of macrophage uptake of oxidized lipids and formation of foam cells [48,52]. Other studies also show that LPS, which is the main substrate for TLR4 recognition, through activation of the receptor signaling pathway induces production of matrix metalloproteinases (MMPs) as well as proteolytic enzymes in macrophages thus contributing to atherosclerotic plaque instability [53].

TLR4 expression is also regulated by oxidized LDL (oxLDL) [54,55,56]. TLR4 is known to be directly involved in the regulation of cholesterol metabolism in macrophages [57]. TLR activation is related to cellular cholesterol levels and can be regulated by reverse cholesterol transport [58,59]. Conversely, TLR activation modifies reverse cholesterol transport. Involvement in cholesterol metabolism may act as an important additional mechanism by which TLR4 may influence atherogenesis.

It is thought that relatively low plasma free fatty acid concentrations may be altered by phospholipases A2 (PLA2) or be related to food intake. In this regard, endothelial cells and blood cells, including monocytes, may be exposed to different concentrations of fatty acids. This seems important given that saturated fatty acids can activate the TLR4 receptor [60] and induce inflammatory responses through this activation [61].

One of the known mechanisms by which saturated fatty acids can activate the TLR4 pathway is that Lipid A, a structural component of lipopolysaccharide (LPS), is acylated with hydroxy saturated fatty acids [62]. Moreover, the 3-hydroxyl groups of these saturated fatty acids can additionally be 3-O-acylated by saturated fatty acids [63]. It is suggested that this structural similarity may be the reason why saturated fatty acids may be involved in TLR4 activation. It should be noted that this mechanism is subject to debate, suggesting a number of other mechanisms [64]. For example, saturated palmitic acid may bind directly to the myeloid differentiation factor 2 (MD2) that is part of the TLR4 complex [65].

Another mechanism that links saturated fatty acids to inflammation is that they can be used to form ceramides, which when incorporated into plasma membranes form lipid rafts that are unique in their biophysical properties and are involved in the activation of signaling pathways associated with inflammation and apoptosis [66]. For example, palmitic acid has been shown to induce sphingomyelin hydrolysis mediated by neutral sphingomyelinase (nSMase), which is involved in IL-6 activation [67].

It was found that saturated palmitic acid can activate pro-inflammatory TLR4 signaling pathways through both MyD88-dependent and MyD88-independent activation of nuclear factor kappa B (NF-kB) [68,69,70]. This leads to increased macrophage production of cytokines such as IL-1β [71,72], tumor necrosis factor alpha (TNF-α) [73], C-C motif chemokine ligand 2 (CCL2)/MCP-1 [68,74], C-C motif chemokine ligand 4 (CCL4)/macrophage inflammatory protein-1 beta (MIP-1β) [75], increased cyclooxygenase (COX)-2 [76] and MMP-9 [71,77]. Palmitic acid also enhances TNF-α production under the influence of LPS [70,72].

Another mechanism that links palmitic acid to inflammation is palmitoylation of proteins involved in the innate immune response [78]. It is assumed that palmitoylation of TLR2 is required for its proper membrane localization and full functional activity [79].

Given that the main product of fatty acid synthesis is palmitic acid, the role of fatty acid synthesis in inflammation may be related to palmitoylation of TLR and the downstream links of its signaling pathway, such as MyD88 [78,80]. Inhibition of fatty acid synthase by C75 specifically inhibits TLR-induced neutrophil activation [80].

Interestingly, TLR4-mediated MyD88- and TRIF-dependent mitogen-activated protein kinase (MAPK) pathways in LPS-induced inflammation, lead to cytosolic phospholipase A2 (cPLA2) activation followed by release of free arachidonic acid and production of proinflammatory lipid mediators [81]. In addition, TLR4 activation leads to the de novo synthesis of fatty acids, which can then be incorporated into plasma membrane sphingolipids [82].

In turn, unsaturated acids are not involved in TLR4 signaling pathway activation. It has been shown that arachidonic acid can bind to MD2 and prevent its association with ligands such as LPS, thus reducing LPS-induced inflammation [83]. The same mechanism involving arachidonic acid can prevent activation of pro-inflammatory TLR4 signaling pathways by saturated fatty acids [83].

Thus, saturated and unsaturated fatty acids may show opposite effects on TLR4 activation [84]. Saturated lauric acid has been shown to induce dimerization and recruitment of TLR4 to lipid rafts. In contrast, polyunsaturated docosahexaenoic acid inhibited lauric acid and LPS induced dimerization and recruitment of TLR4 to the lipid raft fraction, exhibiting anti-inflammatory properties [63,76,85].

In another study, the G-protein-related receptor (GPR) 40 or free fatty acid receptor 1 (FFA1) was shown to be involved in palmitic acid-stimulated IL-6 expression in endothelial cells [67]. FFA1 (GPR40) and FFA4 (GPR120) receptors have been described in macrophages and neutrophils, two key cells mediating the innate immune response [86].

Free fatty acids can also initiate the activation of NLRP3 (NACHT, LRR and PYD domains-containing protein 3) inflammasome [87,88]. In contrast, monounsaturated fatty acids and polyunsaturated fatty acids inhibit NLRP3 activity [89]. This seems important given the role of NLRP3 inflammasome and IL-1β in the pathogenesis of atherosclerosis [90]. The importance of the innate immune system in the development of atherosclerosis is demonstrated by the results of the canakinumab study (CANTOS) showing that anti-inflammatory therapy targeting IL-1β leads to a reduced rate of recurrent cardiovascular events in patients with sustained myocardial infarction, which is not dependent on lipid lowering [91].

## 5. Hemodynamic Characteristics of Blood Flow and Inflammation

**Figure 1:**
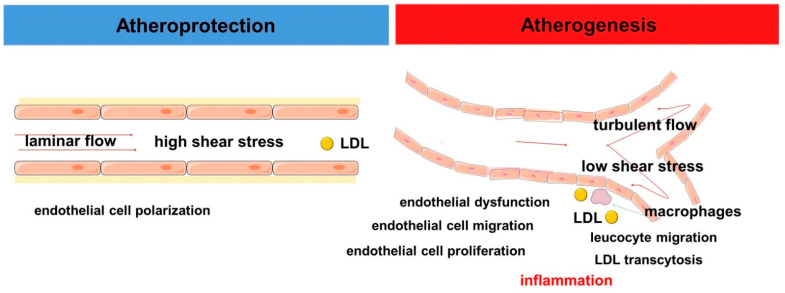
Scheme demonstrating the role of hemodynamics in atherogenesis.

The modern concept of vascular hemodynamics assumes that in the straight parts of arteries the blood flow has a laminar character in which blood moves in orderly layers with a minimum velocity along the vascular wall and a maximum at the geometric center of the vessel. This corresponds to high values of shear stress, i.e., the frictional force that the blood exerts on the vascular wall [92]. This nature of blood flow is considered atheroprotective. In contrast, in the area of curvatures or bifurcations the blood flow has a turbulent character and low shear stress values correspond to it. Areas of arteries that are subject to turbulent blood flow are associated with the development of atherosclerosis. Shear stress is one of the key hemodynamic determinants of endothelial function [93]. Shear stress greater than 15 dyne/cm^2^ has been shown to contribute to an atheroprotective gene expression profile of endothelial cells, with low shear stress (<4 dyne/cm^2^) observed in arterial sites prone to atherosclerosis [94] (Figure 1).

Endothelial cells cover the walls of all blood vessels, including arteries, capillaries and veins and form a barrier that allows adequate hemodynamics to be maintained [95]. Indeed, endothelial cells act in a coordinated manner to detect changes in blood flow and, in turn, can influence it through the production of certain vasoactive mediators [26]. The detection of changes in hemodynamic characteristics of blood flow is complex. It includes some structural and functional changes in the plasma membrane and cellular cytoskeleton organization of endothelial cells in response to shear stress changes [96]. It is also associated with changes in the expression profile of some genes [97,98,99,100,101].

The current paradigm of vascular hemodynamics suggests that the endothelium determines a decrease in the lumen of the vessel and, consequently, a local increase in blood flow velocity and shear stress. In response, the endothelium releases several vasodilatory factors such as nitric oxide, prostacyclin and metabolites formed from arachidonic acid by cytochrome P450 (CYP), which are necessary to relax vascular smooth muscle and regulate arterial lumen [102,103,104,105,106,107].

Shear stress leads to modulation of plasma membrane fluidity through alteration of its lipid composition. This process has complex, not fully understood mechanisms. One such mechanism is the involvement of stearoyl-coenzyme A desaturase 1 (SCD1), an enzyme that catalyzes the desaturation of Δ9-cis saturated fatty acids such as palmitate and stearate, converting them into palmitoleate and oleate [108,109,110]. These unsaturated fatty acids, which are present in membrane phospholipids, can affect the biophysical properties of the membrane by increasing its fluidity. Oleic acid can inhibit endothelial activation by reducing the relative proportions of saturated fatty acids (palmitic and stearic) in total cellular lipids and by reducing VCAM-1 expression and inhibiting NF-κB activation [111].

Laminar flow increases SCD1 expression in endothelial cells via a PPARy-dependent pathway [108]. Studies have shown that SCD1 expression in the endothelium has stable levels in the straight parts of the rat abdominal aorta but decreases in the endothelium on the lateral side of the arterial branch, where the hemodynamic characteristics of the blood flow may differ from the laminar flow [108]. In addition to PPARy activation, shear stress also activates sterol regulatory element-binding protein 1 (SREBP-1) [112,113]. In contrast to laminar flow, perturbed flow causes sustained activation of SREBP1 [112]. Sustained activation of SREBP1 can induce fatty acid synthesis by endothelial cells by enhancing transcription of hydroxymethylglutaryl-CoA (HMG-CoA) synthase and fatty acid synthase genes [112]. In general, known data suggest that unidirectional flow induces fatty acid oxidation, whereas disturbed flow preferentially promotes lipid synthesis and accumulation in endothelial cells, which may affect cell mechanical properties and mechanotransduction [114].

It has also been shown that oscillatory flow can activate SREBP2 and induce NLRP3 inflammasome in endothelial cells. This mechanism is based on the involvement of SREBP2 transactivating NADPH oxidase 2 (NOX2) and NLRP3 [115]. In combination with hyperlipidemia, these cross-links to hemodynamics may be one mechanism for the distribution of atherosclerotic lesions in the vascular bed [115].

Another mechanism that links hemodynamic characteristics of blood flow to the innate immune system is its effect on TLRs. Endothelial cells under laminar flow conditions in an in vitro experiment were less sensitive to TLR2 ligands [49]. Laminar flow can suppress TLR2 expression in endothelium by inducing serine phosphorylation of SP1 via CK2 protein kinase and thereby blocking binding of SP1 to TLR2 promoter, which is necessary for TLR2 expression [49].

In contrast, TLR2 expression in endothelial cells in regions with non-laminar flow was upregulated, especially in the region of the lesser aortic curvature, as shown in an experiment involving low-density lipoprotein receptor–deficient (LDLR^−/−^) mice [116]. Moreover, this regional endothelial TLR2 expression was increased by diet-related hyperlipidemia [116]. This is consistent with evidence that endothelial expression of TLR2 is associated with the development of atherosclerotic lesions in hyperlipidemic mice. TLR2 also induces vascular smooth muscle cell migration into the intima through the formation of IL-6 [117]. Suppression of TLR2 expression is thought to be atheroprotective [49] and may be another mechanism of regional specificity in the progression of atherosclerotic lesions [49].

There are important relationships between shear stress and proinflammatory cytokines such as TNF-α and interleukin IL-1β. In the HUVEC experiment, shear stress has been shown to contribute to suppression of TNF-α signaling via activation of the ERK1/2 signaling pathway, which represents a mechanism for the atheroprotective action of sustained laminar flow [118]. However, no effect on flow-dependent endothelial TLR4 activation has been found [116].

In contrast, the anti-inflammatory transcription factor KLF2 (Kruppel-like factor 2) is activated by high shear stress [93]. KLF2 is involved in the alignment of endothelial cells under shear stress, which is one of the important mechanisms of cellular adaptation to hemodynamic conditions [119]. In addition to hemodynamic flow characteristics, fatty acids associated with a high-fat diet (for example, palmitic acid) may contribute to decreased KLF2 expression in myeloid cells, leading to inflammatory activation [120].

Thus, hemodynamic forces play a fundamental role in the regulation of endothelial cell function and may be associated with innate immune system and inflammation. Given that key risk factors such as dyslipidemia, systemic inflammation and oxidative stress are diffuse, the hemodynamic characteristics of blood flow largely determine the specific localization of atherosclerotic lesions.

## 6. Participation of Fatty Acids in the Regulation of Bioactive Metabolites Related to Hemodynamics and Inflammation

### 6.1. Nitric Oxide

Endothelial cells implement several mechanisms to regulate vascular lumen in response to changes in blood flow. The best-known bioactive substance that ensures vasodilatation is nitric oxide [23], changes in bioavailability of which are the subject of active research in the field of vascular surgery. It should be noted that nitric oxide is also involved in several other vascular functions, such as regulation of platelet and leukocyte adhesion, thrombosis and fibrinolysis [16,121,122]. Nitric oxide is synthesized in the endothelium by a specific nitric oxide synthase (NOS) isoform, also called NOS3. In addition to endothelial nitric oxide synthase (eNOS), several other cell types also express neuronal NOS (nNOS) as well as inducible NOS (iNOS), the latter of which is regulated in response to inflammatory stimuli.

Nitric oxide is essential for the maintenance of normal arterial pressure [123]. Indeed, eNOS overexpression in a mouse model is associated with a reduction in blood pressure (approximately 20 mmHg) and plasma cholesterol levels (approximately 17%), resulting in a 40% reduction in atherosclerotic lesions [124]. Elevated blood pressure levels are of great clinical interest when studying their links to atherogenesis.

Interestingly, however, fatty acids can have different effects on nitric oxide bioavailability [16]. Eicosapentaenoic acid-treated cells show greater NO production while decreasing ONOO- release. Exposure to docosahexaenoic acid increased NO levels by 12% but had no effect on ONOO- release, whereas exposure to arachidonic acid had no significant change in NO and ONOO- release [16]. In turn, both saturated palmitic acid and unsaturated linoleic acid have an inhibitory effect on insulin-stimulated eNOS activation in endothelial cells. Palmitic acid inhibited insulin signaling by promoting PTEN (phosphatase and tensin homolog deleted on chromosome 10) activity, whereas linoleic acid inhibited Akt-mediated phosphorylation of eNOS [125]. In another study, elevated circulating free fatty acid concentrations were shown to suppress eNOS mRNA expression and activity [126]. Indeed, elevated levels of circulating free fatty acids induce endothelial dysfunction and impair endothelium-dependent vasodilation [127].

Thus, free fatty acid-induced reduction of NO production via eNOS may contribute to an increased incidence of arterial hypertension and macrovascular disease in insulin-resistant patients [128].

Palmitoylation is another known mechanism for regulating eNOS activity. Palmitoylation is necessary for the localization of eNOS in caveolae and can regulate NO release [129]. Interestingly, eNOS palmitoylation was reduced in fatty acid synthesis-deficient mice cells, and the incorporation of labeled carbon into eNOS-associated palmitate was dependent on fatty acid synthesis [40].

It should be noted that there is known evidence that eNOS can act as a participant in inflammation [130]. In an experiment in an eNOS-deficient mouse model, the enzyme and the nitric oxide it produces have been shown to play an important role in regulating vascular permeability during the acute phase of inflammation. Deletion of eNOS reduces vascular permeability and tissue edema but is not involved in leukocyte migration to inflamed areas [131]. However, in cutaneous leishmaniasis, NO, produced presumably by eNOS of vascular endothelial cells, counteracted the attraction of granulocytes, thereby limiting the severity of skin lesions [132].

It should be noted that, in the area of advanced atherosclerotic lesions, there is a decrease in eNOS expression by endothelial cells and a significant increase in total NO synthesis by other cell types mainly due to the iNOS isoform. iNOS, which is activated by proinflammatory stimuli including cytokines and LPS, produces significantly more nitric oxide than the eNOS isoform [133,134]. Increased production of NO in the intima may have adverse effects on the arterial wall [135,136,137,138]. This is because, in addition to providing homeostasis, another important function of NO is cytotoxicity. NO produced by iNOS is an important component of macrophage-mediated immune defense against multiple pathogens.

Under physiological conditions, NO is produced in small amounts and mediates vasorelaxation, controls platelet and neutrophil adhesion and aggregation [139,140]. The optimal functional activity of eNOS, which ensures the production of the required amount of NO, is associated with an antiatherogenic effect. Disruption of this balance and increased iNOS activity may be associated with overproduction of NO, which may be involved in lipid peroxidation processes [136,141,142,143,144]. Formed by the interaction of NO and superoxide anion, peroxynitrite is an oxidizing agent that can impair endothelial function [145].

In this regard, another proinflammatory mechanism linking NO and lipid mediators of inflammation should be noted. It has been shown that in insects NO can mediate cellular immunity through the activation of PLA2 and the associated production of pro-inflammatory lipid mediators [146]. Nitric oxide is a small signaling molecule that induces cellular and humoral immune responses through the Toll/Imd signaling pathways in *Drosophila* [147]. In insects, NO signaling is thought to be an upstream component of eicosanoid signaling in response to immune challenge. NO has been shown to mediate hemocytic immune responses by increasing PLA2 activity and eicosanoid biosynthesis, which play an important role in the immune response [146,148].

The concept of cross-talk between NO and eicosanoids signaling has also been described for the mouse macrophage cell line RAW264.7 [149]. It is suggested that NO can enhance COX activity, leading to increased production of pro-inflammatory prostaglandins, and possibly enhancing the inflammatory response [150]. Evidence suggests that NO may interact directly with COX, causing an increase in its enzymatic activity [149,150]. Nitric oxide is thought to regulate prostaglandin synthesis by activating COX-2 through S-nitrosylation. It should be noted that all questions concerning the mechanisms of this interaction have not yet been resolved and remain the subject of discussion [146,150,151,152]. Peroxynitrite at low concentrations induces COX-1 and COX-2 activity, whereas at high concentrations it inhibits the activity of both enzymes [145,153]. Peroxynitrite inactivates COX-1 and COX-2 through tyrosine nitration [150]. Peroxynitrite can induce post-translational modification of COX-2 by affecting an early step in the glycolytic pathway required for N-linked glycosylation of proteins. This leads to a decrease in COX-2 activity and an acceleration of its degradation [145]. These mechanisms may be part of the regulation of prostaglandin synthesis. Given the enzymatic activity of COX and the physiological functions of its metabolites, described in the next chapter, these interactions may be of clinical interest.

Numerous data show that basal NO release and associated endothelium-dependent vasodilation are impaired in hypercholesterolemia [144,154,155,156,157]. For example, an increase in serum LDL levels above 160 mg/dL in patients is accompanied by an impairment of local NO release [158,159]. oxLDLs also impair the balance between constitutive eNOS and inducible iNOS in endothelial cells.

Information about the role of nitro-fatty acids in endothelial function is of interest. These molecules are formed when unsaturated fatty acids react with NO or NO-derived compounds [160,161]. Nitro-oleic acid can increase the bioavailability of NO by increasing eNOS mRNA and phosphorylation of eNOS [162]. In addition, nitro-oleic acid modulates endothelin signaling by causing an Nrf2-dependent increase in endothelin B (ET-B) receptor expression in endothelial cells. This leads to a decrease in the extracellular concentration of endothelin-1 (ET-1), thereby limiting its vasoconstrictor effects [161]. Thus, the biological activity of nitro-fatty acids is associated with endothelial vasorelaxation, inhibition of platelet aggregation, and anti-inflammatory effects [163,164].

It is suggested that the formation of nitrated lipid compounds can be considered as a form of NO storage, which acts as a compensatory mechanism of impaired endothelium-dependent vasorelaxation in the early stages of vascular disease [163].

Thus, the regulation of nitric oxide synthesis and NO bioavailability appear to be important for the development and progression of atherosclerosis. At the same time, fatty acids may be involved in the regulation of NO production and functional activity.

### 6.2. Eicosanoids

Bioactive lipids, which are metabolites of fatty acids, are involved in many inflammation-related processes and may be actively involved in the pathogenesis of atherosclerosis. The most active precursor of eicosanoids is arachidonic acid, which is part of the phospholipids of plasma membranes.

It is known that shear stress can activate cPLA2, which promotes the release of arachidonic acid from phospholipids of the plasma membrane [165]. Phospholipases A2 is a family of enzymes that hydrolyze the acyl bond at the sn-2 position of phospholipids to form free fatty acids and lysophospholipids [166]. Arachidonic acid produced by PLA2 is metabolized to form eicosanoids, which have a variety of physiological and pathophysiological effects [166,167].

**Figure 2:**
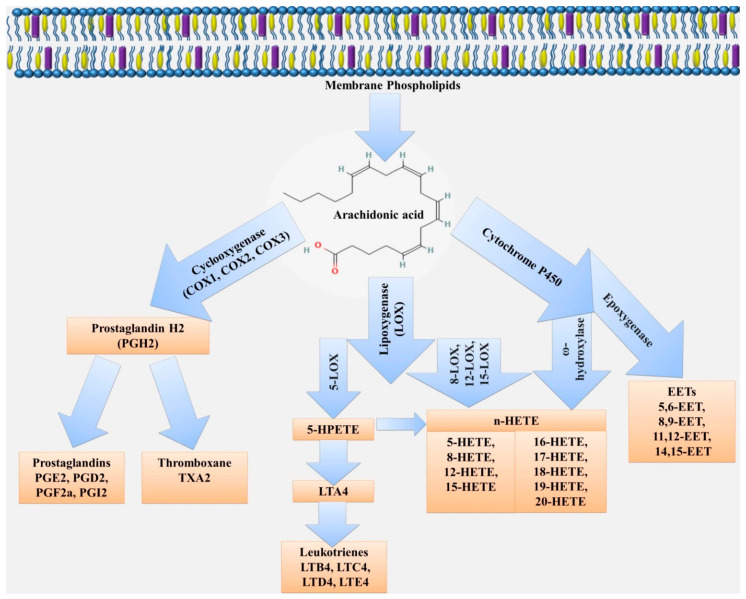
Scheme of eicosanoid biosynthesis from arachidonic acid.

Arachidonic acid can be metabolized by three major enzymatic pathways: COX, lipoxygenase (LOX) and CYP. The enzymatic conversions produce a variety of different, and sometimes opposing, products in function and activity [168,169]. These metabolites may affect the function of both endothelial and other cells in the vascular wall. For example, the COX pathway converts arachidonic acid to produce prostaglandins (PGE2, PGF2, PGD2), thromboxane (TXA2) and prostacyclin (PGI2). The lipoxygenase pathway promotes the formation of LT, lipoxins, resolvins and other metabolites [18,170,171,172]. CYP enzymes contribute to the formation of hydroxyeicosatetraenoic acids (16-, 17-, 18-, 19- and 20-HETE) and epoxyeicosatrienoic acids (EETs; 5,6-EET, 8,9-EET, 11,12-EET and 14,15-EET) (Figure 2).

#### 6.2.1. Cyclooxygenase Pathway of Arachidonic Acid Metabolism

Arachidonic acid is converted by the enzyme cyclooxygenase into prostaglandin G2 (PGG2), which is metabolized to prostaglandin H2 (PGH2) by peroxidase, whose further metabolism to bioactive products is dependent on different enzymes. PGF2α reductase promotes the synthesis of PGF2α, PGE2 synthase promotes the synthesis of PGE2, PGI2 synthase promotes the synthesis of PGI2, and TXA2 synthase converts PGH2 into TXA2 [172,173,174,175,176]. In addition, prostaglandins can be converted into each other by enzymatic conversion, e.g., PGE2 and PGF2α can be synthesized from one another by the enzyme aldo-keto reductase (AKR) 1C1 and AKR1C2 [177].

PGI2 is a major prostanoid that is produced in the endothelium of large vessels and vascular smooth muscle cells and influences the IP receptor, which helps to relax vascular smooth muscle and leads to a pronounced vasodilator effect as well as suppression of platelet aggregation. Thus, prostacyclin has an anti-inflammatory effect and prostacyclin analogues are used to treat pulmonary hypertension [178,179,180,181,182]. In addition, PGI2 regulates the immune system with an immunosuppressive effect [179]. Reduction of PGI2 in the endothelium can promote lipid deposition and the development of atherosclerosis [179,183]. Blood pressure and shear stress modulation significantly increase PGI2 formation, with changes in blood pressure playing a key role in this process and may be the cause of atherosclerosis [184].

Depending on the cell types, certain products predominate among metabolic products. When arachidonic acid is metabolized in platelets, the main metabolite will be TXA2, which has a pro-aggregative effect, and in the vascular endothelium, prostacyclin will be predominantly produced, which, conversely, will have an anti-aggregative effect. Macrophages produce PGE2 and TXA2, and mast cells generate PGD2 [185].

Some pathological conditions alter the formation and ratio of arachidonic acid metabolic products. For example, in atherosclerosis there is a decrease in prostacyclin synthesis and an increase in TXA2, this will contribute to increased inflammation, disease progression or an increased risk of thrombosis [168,169,178]. In addition, cell activation will alter the composition and activity of enzymes, which will also lead to changes in the synthesis of arachidonic acid metabolic products [186,187].

In vascular smooth muscle cells, induction of COX-2 has been shown to result in increased production of PGE2 [188,189,190,191]. PGE2 acts on EP1, EP2, EP3, EP4 receptors [192,193]. Activation of EP1 and EP3 produces vasoconstriction and blood pressure regulation [194], whereas activation of EP2 and EP4 receptors produces vasodilation and a decrease in blood pressure [194]. It is the effect of PGE2, more so on EP1 receptors, that plays a key role in the pathogenesis of many diseases [190,195,196].

Most studies confirm the important role of PGE2 in the pathogenesis of atherosclerosis [190,197,198,199]. The mechanism is related to the marked activation of COX-2 in macrophages, resulting in the activation of chemotaxis, the production of inflammatory cytokines, mainly PGE2, which contributes to changes in vascular permeability and stimulates the migration of vascular smooth muscle cells [200]. Elevated PGE2 levels are one of the most important markers, along with C-reactive protein (CRP), IL-6, VCAM-1, myeloperoxidase, secretory PLA2 and COX-2, indicating high risk of myocardial infarction and associated with poor prognosis [201,202,203].

PGF2α is an unstable compound, but its active metabolite 5-keto-dihydro-PGF 2α has better stability. PGF2α exerts its action via the prostaglandin F2-alpha receptor (PGF), also known as prostanoid FP receptor (FP) [204], which has two subtypes FPA and FPB. FP expression is found in many organs and tissues, including the cardiovascular system and is involved in the pathogenesis of coronary heart disease. It has been established in the experiments in vitro that PGF 2α is formed in large amounts in endothelial cells in response to shear stress and can affect blood pressure [205,206]. These data make the study of the effect of FP receptor blockers in the comorbid course of arterial hypertension and atherosclerosis promising [206,207].

TXA2 is largely produced by platelets as well as monocytes and acts via the thromboxane A2 receptor, also known as Prostanoid TP receptor (TP), which is involved in many processes, including atherogenesis [178,208,209,210]. The functions of TXA2 are not only platelet activation, it also has a significant vasoconstrictor effect [211] and a mitogenic effect on the vascular smooth muscle cells through its effect on TP and activation of the YAP/TAZ protein [212]. This effect of TXA2, in combination with other factors, could be linked to the development of atherosclerosis, especially as TXA2 production is significantly increased in atherosclerosis [183,213,214].

In addition, isoprostanes (IsoPs), products of arachidonic acid peroxidation, which are prostaglandin isomers by chemical structure, exert their effect through TP receptors [178,213,215,216]. There are distinguished F2-isoprostanes (F2-IsoPs), the main representatives of this group are 8-epi-PGF2α, IPF2α-I, which are also found in atherosclerotic plaque [215]. Thus, IsoPs are of interest not only as prostaglandin analogues but also as biomarkers that can potentially be used as indicators of cardiovascular diseases such as coronary heart disease and atherosclerosis and oxidative stress [217]. This is because IsoPs are among the most sensitive and reliable representative markers of peroxidation [218,219,220,221,222,223]. In addition, the amount of 8-IsoP is significantly elevated in respiratory diseases such as COPD [224], asthma [225,226,227,228] and others [229,230,231,232], which is an important marker of the comorbid course [221].

In the walls of large arteries an unstable compound prostaglandin X (PGX) is formed from arachidonic acid or prostaglandin endoperoxides PGG2 or PGH2, which relaxes the vascular smooth muscle and promotes the expansion of the vascular lumen and the reduction of platelet aggregation [233].

There is some evidence of a direct link between NOS, COX and CYP4A enzymes [234,235,236,237]. NO has been shown to activate cyclooxygenase enzymes, resulting in increased prostaglandin formation [236,237]. These data indicated the possibility that COX enzymes could be considered as targets for modulating the role of NO in inflammation [236].

#### 6.2.2. Lipoxygenase Pathway

The lipoxygenase pathway contributes to the formation of a number of important metabolites involved in both the development and resolution of inflammation. The human genome contains 6 functional LOX genes (*ALOX5, ALOX12, ALOX12B, ALOX15, ALOX15B, ALOXE3*) encoding 6 different LOX isoforms [238,239]. ALOX5 and ALOX15 are considered to be the most characteristic human isoenzymes [238].

The ALOX5-related leukotriene signaling pathway is an evolutionarily ancient inflammatory mechanism present in all higher vertebrates [171,240]. High levels of expression of the 5-lipoxygenase pathway have been found in the arterial walls of patients suffering from atherosclerosis, resulting in the formation of leukotrienes, which have strong pro-inflammatory activity [241]. Leukotriene B4 (LTB4) plays a key role in inflammatory reactions by enhancing the expression of endothelial adhesion molecules and promoting leukocyte transmigration and activation. Enhancement of monocyte chemotaxis by CCL2 production and conversion of monocytes to foam cells by enhancing CD36 expression and fatty acid accumulation is one of the mechanisms of atherogenesis involving LTB4 [242]. LTB4 is associated with both onset and progression of atherosclerosis, including the development of a vulnerable plaque phenotype and adverse clinical outcome [243]. Simultaneously with high LTB4 levels, specialized pro-resolving mediators (SPMs) levels, especially Resolvin D1 (RvD1), and the SPM to LTB4 ratio decrease in the region of unstable atherosclerotic plaques [244].

15-lipoxygenase (ALOX15) has attracted much research attention for its role in promoting active resolution of the inflammatory process. It is a key enzyme in the synthesis of SPM [245].

It should be noted that the oxygenation reaction catalyzed by 15-lipoxygenase converts arachidonic acid to 15-hydroperoxyeicosatetraenoic acid (15-HPETE), which is then metabolized to 15-hydroxyeicosatetraenoic acid (15-HETE). 15-HETE is a potent vasoconstrictor, contributing to pulmonary hypertension [246]. 15-HETE may play a role in atherogenesis because it is a chemotactic agent for vascular smooth muscle cells and mitogenic for endothelial cells. It is also an inhibitor of prostacyclin synthesis [247].

In addition, ALOX15 and ALOX15B can oxidize esterified PUFAs in membranes, lipoproteins and cholesterol esters and may promote foam cell formation [245,248,249,250]. However, these data should be interpreted with caution, given the diverse function of ALOX15 lipid products also involved in the resolution of inflammation.

#### 6.2.3. Cytochrome P450 Pathway

Of the large CYP family of enzymes, which contains many subclasses, the ω-hydroxylase and epoxygenase enzymes are the most important for arachidonic acid metabolism. The ω-hydroxylase activity of the CYP enzymes leads to the formation of hydroxyeicosatetraenoic acids (16-, 17-, 18-, 19- and 20-HETE). The epoxygenase activity of CYP enzymes is linked to the formation of arachidonic acid epoxides or epoxyeicosatrienoic acids (EETs; 5,6-EET, 8,9-EET, 11,12-EET and 14,15-EET) known as endothelium-derived hyperpolarizing factors [172].

One of the best-known members of the HETE group is 20-HETE, which has pro-inflammatory effects and is associated with vasoconstriction. Because of its vasoconstrictor effect, 20-HETE may be involved in the regulation of blood pressure [235]. In addition, 20-HETE is involved in vascular remodeling and promotes endothelial dysfunction and angiogenesis by increasing the expression of VEGF and vascular endothelial growth factor receptor 2 (VEGFR2) [235,251]. 20-HETE induces endothelial dysfunction by inhibiting the association of eNOS with the 90 kDa heat shock protein (HSP90) [252,253,254]. Meanwhile, NO can reduce 20-HETE production by inhibiting CYP ω-hydroxylase activity [234,235,254].

The role of 20-HETE in inflammation may be related to the fact that it promotes endothelial activation by stimulating NF-kB and increasing the levels of pro-inflammatory cytokines such as IL-8 and adhesion molecules (ICAM/VCAM) [255,256,257]. 20-HETE can bind to G-protein receptor 75 (GPR75), activating a number of signaling pathways and inducing endothelial dysfunction [258,259] as well as vascular smooth muscle cell migration and hypertrophy [256].

The main source of 20-HETE in the vascular wall is vascular smooth muscle cells [256,260]. Other cells with which 20-HETE production may be associated are myeloid cells, neutrophils and platelets [256,261,262].

In contrast, 5,6-, 8,9-, 11,12- and 14,15-EET, have vasodilatory and anti-inflammatory activity [172]. However, in addition to regulating vascular tone, EETs also affect endothelial cell and vascular smooth muscle cell proliferation and migration, extracellular matrix degradation and angiogenesis [263,264,265].

Thus, lipid mediators derived from arachidonic acid play a central role in immune regulation, and their role in vascular biology and pathogenesis of atherosclerosis seems very important.

## 7. Conclusions

Atherosclerosis is a chronic multifactorial disease, the development and progression of which are associated with an imbalance of pro- and anti-inflammatory factors against the background of lipid metabolism disorders. Endothelial cells are at the intersection of many pathways, providing regulation of hemodynamic characteristics of blood flow, regulation of innate immune response, including by influencing the activity of other cells.

**Figure 3:**
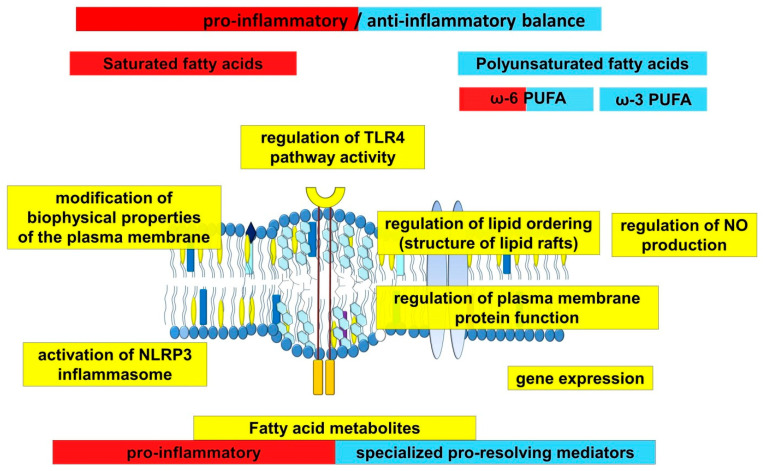
Scheme demonstrating the role of long-chain fatty acids in inflammation.

Fatty acids play an important role in atherogenesis, performing not only a structural or energetic function, but also being participants in many processes related to the regulation of hemodynamics and the innate immune system (Figure 3). Their function is multifaceted and related to their chemical structure, demonstrating the differences for saturated and unsaturated fatty acids. This information underlies many therapeutic strategies in the treatment of atherosclerosis, which include dietary adjustments and intake of ω-3 PUFAs.

Given the role of fatty acids in the pathogenesis of atherosclerosis, the data on the existence of cross-linkages between fatty acids and some drugs used to treat patients with atherosclerosis seem to be important.

It is known that statin therapy can reduce the concentration of free fatty acids in blood plasma [266]. It has been shown that taking rosuvastatin led to a significant decrease in the concentration of saturated and monounsaturated fatty acids in the free fatty acid fraction. However, these changes did not affect long-chain polyunsaturated fatty acids with 20 and 22 carbon atoms [267].

Statins and PUFAs are thought to have some similar anti-inflammatory actions, such as inhibition of proinflammatory cytokine IL-6 and TNF-α production and NF-kB activation. They also increase endothelial nitric oxide synthesis, thereby exerting anti-atherosclerotic effects [268,269,270,271,272,273].

The association of fatty acids with angiotensin-converting enzyme (ACE) is interesting. Free fatty acids caused angiotensin II-dependent activation of leukocytes, which impaired endothelial function by increasing myeloperoxidase release and, presumably, increasing leukocyte adhesion [274]. In turn, inhibition of the renin-angiotensin system prevents acute endothelial dysfunction induced by free fatty acids [275]. Thus, ACE inhibitors or angiotensin II receptor blockers (ARBs) can prevent endothelial dysfunction induced by free fatty acids. At the same time, long-chain PUFAs inhibit ACE activity and reduce angiotensin II formation [276].

Aspirin is known to be involved in the biosynthesis of lipid mediators associated with the resolution of inflammation, which may play an important role in antiatherogenic action [277]. Circulating aspirin-triggered lipoxin levels have been shown to inversely correlate with the severity of peripheral artery disease [278]. These data underscore a potential therapeutic opportunity for resolution-promoting lipid mediators in controlling vascular inflammation and atherogenesis. In general, SPMs are of great interest as promising therapeutic agents.

Thus, the development of atherosclerosis is closely related to the imbalance of pro- and anti-inflammatory mechanisms. Fatty acids are involved in several key functions of endothelial cells and the dysregulation of these processes is closely associated with the development and progression of atherosclerosis.

## Data Availability

Not applicable.
